# Traffic Light Recognition Assistant for Color Vision Deficiency Using YOLO with Multilingual Audio Feedback

**DOI:** 10.3390/s26041093

**Published:** 2026-02-08

**Authors:** Yinyuan Ma, Fathan Arifah, Qonita Afifah, Liko Bun, Kangfu Zhang, Minan Tang

**Affiliations:** 1 School of Mechatronic Engineering, Lanzhou Jiaotong University, Lanzhou 730070, China; mayy@mail.lzjtu.cn (Y.M.); 12251513@stu.lzjtu.edu.cn (K.Z.); 2 Fakultas Teknologi Industri, Universitas Ahmad Dahlan (UAD), Yogyakarta 55191, Indonesia; qonita2000018293@webmail.uad.ac.id; 3 College of International Student Education, Chongqing Medical University, Chongqing 400016, China; likobun@stu.cqmu.edu.cn; 4 School of Automation and Electrical Engineering, Lanzhou Jiaotong University, Lanzhou 730070, China; tangminan@mail.lzjtu.cn

**Keywords:** color vision deficiency, traffic light recognition, spatial-position inference, intelligent transportation systems, assistive sensing technology

## Abstract

Drivers with color vision deficiency (CVD) often face difficulty recognizing traffic light colors at intersections. Relying solely on their limited color vision can increase safety risks while driving in urban environments. In the era of technological development, Intelligent Transportation Systems (ITSs) increasingly aim to provide support for traffic users, including individuals with CVD. To address user needs from diverse backgrounds, this study aims to develop a traffic light recognition system that provides offline multilingual audio feedback in Indonesian, Mandarin, and English. The proposed approach introduces a spatial-position inference framework by applying a full-frame traffic light annotation strategy to a YOLOv12 model, enabling traffic light state recognition based on the relative position of active lights rather than relying primarily on color information. This work contributes to reducing reliance on color-based perception traffic signal recognition frameworks tailored for assistive ITS applications targeting users with color vision deficiency. System performance is evaluated to verify its feasibility using a comprehensive dataset consisting of various traffic light conditions, including daytime and nighttime scenarios, varying weather, and different traffic densities. Experimental results show an average detection confidence of approximately 0.73, with a maximum confidence of 0.95 and low processing latency of 0.214 s on a CPU-only configuration. The system has the potential to enhance driving safety for individuals with color vision deficiency by offering an additional intelligent assistive tool instead of replacing standard driving regulations.

## 1. Introduction

Traffic lights are critical traffic control devices that regulate the movement of road users at intersections and play a key role in reducing conflicts and preventing accidents. Traffic safety is strongly dependent on drivers’ ability to accurately interpret traffic signals. However, for people with color vision deficiency (CVD), commonly known as color blindness [1], relying solely on their limited visual color perception can be challenging when driving. CVD affects approximately 8% of the male population and 0.5% of the female population worldwide [2]. Clinically, the main difficulty lies in the failure of cone cells in the retina to distinguish between certain wavelengths of light, particularly between the red and green spectrums. The impact is not limited to comfort, but also to the ability to participate safely in modern mobility [3]. Research shows that most color-blind people have difficulty driving a vehicle and distinguishing colors on traffic lights under certain conditions. In bright sunlight, red lights look dim and fail to stand out against the background of sky, trees, and buildings [4]. This is exacerbated by the fact that overall visual impairment correlates directly with an increase in traffic accidents, especially in densely populated urban areas with high levels of visual pollution [5]. Beyond navigation, poor color discrimination also hinders an individual’s ability to interpret scientific data or spatial information, necessitating inclusive color standardization [6,7].

Regulations regarding driver’s license ownership are highly inconsistent globally. In the ASEAN region, countries such as Indonesia, Cambodia, and Thailand enforce very strict rules through the Ishihara test [8]. In many cases, this test acts as an absolute barrier to obtaining a driving license, despite ongoing academic debate regarding the direct relationship between CVD and fatal traffic accidents [9]. Research in the European context suggests that color vision impairment does not pose a significant threat to traffic safety, leading many countries in Europe, Canada, and Oceania to eliminate specific color vision standards for private drivers [10]. In the United Kingdom, for instance, the government explicitly states that CVD is not a notifiable condition, and drivers are permitted to drive without medical consultation as long as they meet standard visual acuity and field requirements [11].

Due to inconsistencies in traffic regulations across countries and the inherent limitations of existing traffic light detection systems, safety risks remain a critical issue for transportation users, particularly individuals with color vision deficiency (CVD). The UN Convention on the Rights of Persons with Disabilities (CRPD) is the most widely ratified human rights convention, affirming the right to participation in society on an equal basis with others [12]. Although Intelligent Transportation Systems (ITSs) have evolved rapidly to enhance traffic efficiency and safety, most current implementations are not fully inclusive and often overlook the specific needs of vulnerable user groups. Assistive technologies designed for the disabilities community are still limited in availability [13], including the color-blind sufferer, lack multilingual support, and are unevenly implemented, especially in developing countries, where regulatory conditions and infrastructure development are not yet uniform. Meanwhile, recent research efforts have largely focused on autonomous driving technologies intended for highly developed transportation environments [14]. In response to these gaps, this study proposes a reliable ITS-based assistive tool that provides multilingual audio feedback for traffic signal recognition, to support safer and more accessible mobility for color-blind users living in regions with different regulatory environments and socio-economic backgrounds. Unlike autonomous driving systems, the proposed approach is explicitly designed as an assistive aid that supports human drivers without replacing driving judgment or regulatory responsibility.

To better understand the limitations of existing technical approaches and how they relate to these challenges, the following section reviews prior work on traffic light recognition and assistive vision systems. The remainder of this paper is organized as follows. Section 2 discusses related work in the field of traffic light recognition and visual assistance systems, particularly for drivers with color vision deficiency. Section 3 describes the proposed framework, including a full-frame annotation strategy and the selection of a system architecture suitable for application-level implementation. Section 4 presents the experimental setup and evaluation results under various real-world conditions, focusing on system performance, detection confidence, and processing latency in a CPU-based configuration. Finally, Section 5 discusses the system implementation and user interface design, and Section 6 concludes the paper by summarizing the main contributions and outlining future research directions.

## 2. Related Works

### 2.1. Deep Learning-Based Traffic Light Recognition

Deep learning-based approaches have been widely adopted for traffic light recognition due to their ability to automatically learn discriminative visual features and achieve real-time performance in complex urban environments. Early assistive systems for drivers with color vision deficiency relied on traditional image processing techniques such as RGB adjustment, thresholding, and filtering, which were highly sensitive to illumination changes and weather conditions [4]. Subsequent advances introduced convolutional neural networks for traffic light detection, tracking, and classification, demonstrating improved robustness and accuracy, even for small or distant signals [15,16,17]. Comprehensive surveys indicate that most existing methods can be categorized as modified generic object detectors, multi-stage pipelines, or task-specific single-stage networks, all of which achieve strong performance under controlled conditions but degrade under motion blur, nighttime illumination, or adverse weather [18]. To mitigate these challenges, several studies have explored lightweight architectures for embedded platforms [19,20], motion blur restoration techniques [21], and the integration of prior maps to identify relevant traffic lights along predefined routes [22]. More recent works further enhance detection accuracy by optimizing YOLO-based architectures and incorporating temporal modeling to recognize both static and flashing light states [23,24,25]. While these approaches demonstrate high detection precision and real-time capability, they remain primarily object-centric and implicitly rely on color separability or appearance consistency, assumptions that limit their effectiveness as assistive tools for drivers with color vision deficiency, particularly in visually degraded or low-contrast conditions.

### 2.2. Color-Based and Image Processing Approaches for CVD

Color-based and image processing approaches have been widely explored to assist individuals with color vision deficiency by enhancing color separability or explicitly labeling color information in images. Several studies propose image re-coloring or enhancement techniques to remap confusing color channels into more distinguishable representations, demonstrating improved color discrimination in static natural images under controlled conditions [26]. Other works focus on pixel-level color detection using RGB or HSV thresholding to identify dominant colors and display their corresponding names or values, often implemented through OpenCV-based pipelines [27,28,29,30]. These systems are primarily designed for general color identification tasks, such as recognizing object colors or assisting with daily activities, rather than safety-critical driving scenarios. Although some approaches integrate convolutional neural networks to detect colored objects and report pixel-level color attributes [31], the underlying decision process remains fundamentally dependent on color intensity and chromatic consistency. As a result, these methods are highly sensitive to illumination changes, glare, shadows, weather conditions, and nighttime lighting, all of which frequently occur in real-world traffic environments. Critically, such color-centric strategies replicate the same perceptual limitation experienced by drivers with color vision deficiency, making them unsuitable for reliable traffic light recognition, where false positives or false negatives can directly compromise driving safety. Consequently, purely color-based or pixel-level image processing approaches lack the robustness and structural awareness required for assistive traffic light recognition systems intended for CVD drivers.

### 2.3. Voice-Assisted Vision Systems

Voice-assisted vision systems have been increasingly explored to improve accessibility for visually impaired users by integrating object detection with audio feedback mechanisms [32,33,34]. Several studies employ convolutional neural networks to recognize traffic signs or general objects and convey the detection results to users through text-to-speech narration, enabling hands-free interaction and real-time awareness of the surrounding environment. Other works extend this paradigm to general object detection for blind or visually impaired individuals, using YOLO-based architectures combined with speech synthesis to announce detected objects and their relative positions [35,36]. While these systems demonstrate the practical value of audio feedback in assistive applications, they are predominantly designed for generic object awareness or pedestrian navigation rather than safety-critical driving scenarios. In particular, most approaches do not focus on traffic light recognition as a distinct problem, nor do they explicitly address the perceptual challenges faced by drivers with color vision deficiency. The detected objects are typically treated independently, without modeling the structural or spatial configuration of traffic signals that is essential for interpreting traffic light states. Moreover, several systems rely on online services or cloud-based text-to-speech engines, which may limit reliability in real-world driving conditions. Consequently, although voice-based assistive systems provide an important foundation for accessibility, existing solutions lack traffic-light-specific reasoning and CVD-aware design, highlighting the need for an assistive framework that combines real-time traffic light state inference with fully offline and structurally informed audio feedback.

### 2.4. Research Gap and Rationale

Inspired by prior studies on traffic light recognition and assistive vision systems, this work addresses a practical gap in intelligent transportation research related to the support of drivers with color vision deficiency (CVD). Although traffic light recognition has been widely studied, most existing approaches are designed either for autonomous vehicles or rely heavily on color-based perception, which poses inherent limitations for users with impaired color discrimination. The core contribution of this study lies in reframing traffic light state recognition from a color-centric detection task into a spatial-position inference problem that explicitly leverages the physical structure of traffic lights. By focusing on the relative position of active lights within a full traffic light frame, this work aims to reduce dependence on color perception while maintaining real-time feasibility.

To this end, rather than proposing a new detection algorithm, this study introduces a system-level assistive framework that integrates a full-frame traffic light annotation strategy with a practical deep learning backbone and user-centered feedback design. The proposed approach encodes structural constraints of traffic lights during training and inference, enabling the system to infer signal states based on spatial configuration instead of purely chromatic cues. This framework is implemented as a real-time assistive prototype with a graphical user interface and fully offline multilingual audio feedback, designed to operate on a CPU-only platform. The system is evaluated under diverse real-world conditions, including variations in lighting, weather, and traffic density, to assess its feasibility and responsiveness as an assistive tool rather than a decision-making replacement.

The main contributions of this paper can be summarized as follows:

(1) We reformulate traffic light recognition for drivers with color vision deficiency as a spatial-position inference problem that minimizes reliance on color-based perception.
(2) We introduce a full-frame traffic light annotation strategy that encodes the structural and positional relationships of signal lights to support robust state inference.
(3) We design and implement a real-time assistive traffic light recognition prototype with a PyQt5 graphical user interface and fully offline multilingual audio feedback in Indonesian, Mandarin, and English.
(4) We evaluate the feasibility of the proposed system under diverse real-world conditions, reporting detection confidence and processing latency on a CPU-only configuration.

## 3. Proposed Methods

This section describes the proposed assistive framework for traffic light recognition designed for drivers with color vision deficiency (CVD). The framework emphasizes structural and spatial-position inference rather than color-centric perception, aligning with the limitations identified in existing approaches. The system is designed as an assistive tool that supports human drivers by providing reliable traffic light state information through offline audio feedback, without replacing driving decisions or regulatory responsibility.

### 3.1. Framework Overview and Design Principles

The proposed system follows a two-phase framework consisting of an offline model training phase and a real-time on-device inference phase. Unlike conventional traffic light recognition approaches that rely primarily on color discrimination, this study treats the entire traffic light as a single structural entity. Traffic light states are inferred based on the spatial position of the active signal within the physical frame (top, middle, or bottom), which remains consistent across lighting and environmental variations.

**Figure 1:**
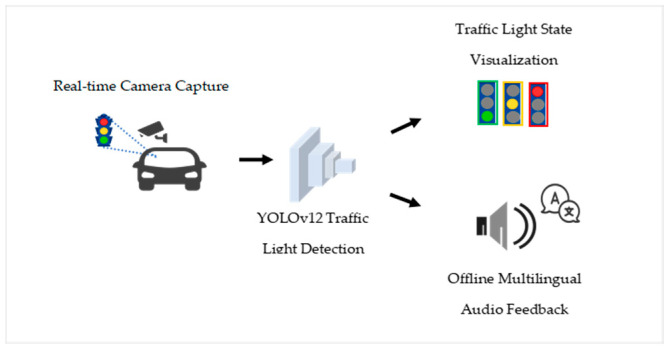
Workflow of the proposed assistive traffic light recognition framework.

In the first phase, a deep learning-based object detection model is trained using annotated traffic light images that encode structural consistency across different signal states. In the second phase, the trained model weights are deployed in a real-time assistive system that processes live video input and generates traffic light state predictions. These predictions are subsequently communicated to users through multimodal feedback, including visual indicators and offline multilingual audio output. The overall workflow of the proposed system is illustrated in Figure 1.

### 3.2. Dataset Preparation and Annotation Strategy

The dataset was collected using camera video recordings taken at locations in various urban areas in Indonesia and China, which were converted into individual image frames using a video-to-frame extraction process. The extracted images were manually annotated using Label Studio v1.14.0 (HumanSignal Inc., San Francisco, CA, USA), where bounding boxes were applied to the entire traffic light structure following the proposed full-frame annotation strategy. Each annotated sample was assigned a corresponding traffic light state label based on the active signal position.

In total, approximately 1000 annotated images were collected, with each traffic light state category containing between 200 and 600 samples. The annotated dataset was exported in the standard YOLO format, consisting of paired image and label files. For model training and validation, the dataset was randomly split into 80% training data and 20% validation data. The training process was conducted to obtain a practical detection model for subsequent system evaluation, rather than to optimize detection performance exhaustively.

### 3.3. Model Custom Training and Performance Comparison

**Figure 2:**
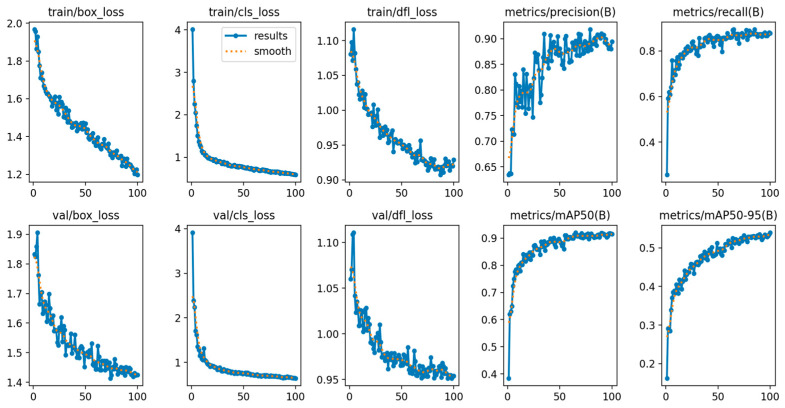
YOLO12 Model Custom Pre-trained Results.

Model training run on a system equipped with an Intel Core i7-3770 processor operating at 3.40 GHz, running on Windows 11 (Microsoft Corporation, Redmond, WA, USA). The model was trained for 100 epochs, with approximately 125 batches per epoch. The number of batches per epoch is determined by dividing the training dataset size by a batch size of 8. Additional training settings include an input image dimension of 640 × 640 pixels, an initial learning rate of 0.01 with linear decay, and a Stochastic Gradient Descent (SGD) optimizer with momentum of 0.937. During the training process, the box loss, classification loss, and distribution focus loss gradually decreased from the initial epoch to the 100th epoch, as shown in Figure 2.

Evaluation results for model training demonstrated a mean Average Precision (mAP@0.5) between 0.92 and 0.95 at completion of training, whereas mAP@0.5:0.95 varied from 0.53 to 0.54. The precision at the 100th epoch attained a range of 0.89 to 0.91, while the recall achieved a range of 0.87 to 0.88. Various other YOLO models from Ultralytics YOLO v8.3.27 (Ultralytics Inc., Fredrick, MD, USA), including YOLO 8n, YOLO 10n, YOLO 11n, and YOLO 12n, were also trained to ascertain optimal performance on the identical dataset, with implementation based on PyTorch v2.5.1 (Meta Platforms, Inc., Menlo Park, CA, USA). The training outcomes for each model are displayed in Table 1. YOLO 11n attained the best precision at 0.927 and recall at 0.895, although YOLO 12n exhibited the highest mAP@0.95 at 0.539. Simultaneously, YOLO 10n demonstrated the lowest performance among the four models, achieving a recall of 0.840.

Based on the results presented in Table 1, YOLOv11n and YOLOv12n demonstrate comparable performance in terms of precision, recall, and mAP, indicating that both models are suitable candidates for traffic light detection under the given dataset constraints. While YOLOv11n achieves slightly higher precision and recall, YOLOv12n attains the highest mAP@0.5:0.95, suggesting better overall localization robustness across varying detection thresholds. Considering the safety-critical nature of assistive traffic light recognition, model selection is not solely based on aggregate accuracy metrics but also on the balance between false positives and false negatives. Therefore, a deeper analysis using confusion matrices is conducted in the following subsection to justify the final model choice for real-time assistive deployment.

### 3.4. Model Selection Rationale

**Figure 3:**
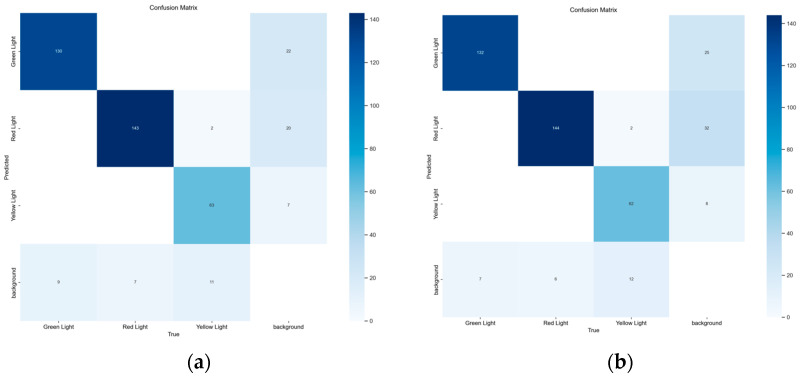
Comparison of YOLO 11n and YOLO 12n; (**a**) YOLO11 Confusion Matrix; (**b**) YOLO12 Confusion Matrix.

Although multiple YOLO variants demonstrated competitive performance, final model selection was based on a detailed analysis of detection stability and error characteristics. Confusion matrix analysis was performed for YOLOv11n and YOLOv12n, as shown in Figure 3.

YOLO 11n and YOLO 12n are suitable for use in detection systems, both showing a balance between precision, recall, and mAP. The selection of YOLOv12 as the final model is based on the confusion matrix analysis shown in Figure 3. YOLO 11n produces 25 false negatives, while YOLO 12n produces 27 false negatives, indicating that YOLO 11n misses fewer objects that actually exist. YOLO 11n also returns a higher number of false positives in the background, with 65 backgrounds incorrectly detected as objects compared to YOLO 12n’s 49. YOLOv12 offers consistency in distinguishing between valid objects and background areas, including minority categories such as yellow lights. Given the traffic application requirements, we prioritize a balance between sensitivity and stability. Although YOLOv12 has a marginally higher false negative rate (a difference of only 2 instances), it significantly reduces false positives (by 16 instances). Minimizing false positives is crucial to prevent driver distraction and ‘alarm fatigue,’ ensuring the driver continues to trust and use the assistive system.

## 4. Experimental and Results Analysis

This section presents an evaluation of the proposed system, assessing detection accuracy across diverse scenarios. The analysis is categorized into performance metrics and environmental robustness tests to verify the system’s reliability for drivers with CVD.

### 4.1. Environmental Scenario Setup

**Figure 4:**
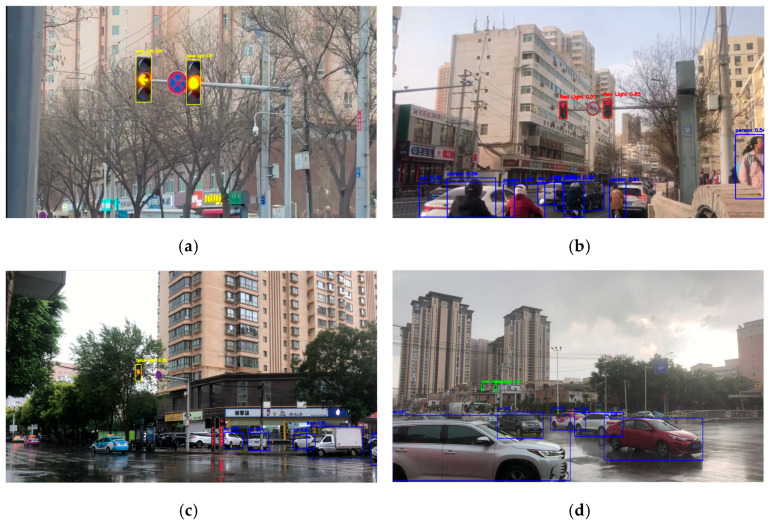
Daytime testing scenarios. (**a**) Clear weather, Normal traffic (DCN); (**b**) Clear weather, Crowded traffic (DCC); (**c**) Rainy weather, Normal traffic (DRN); (**d**) Rainy weather, Crowded traffic (DRC).

**Figure 5:**
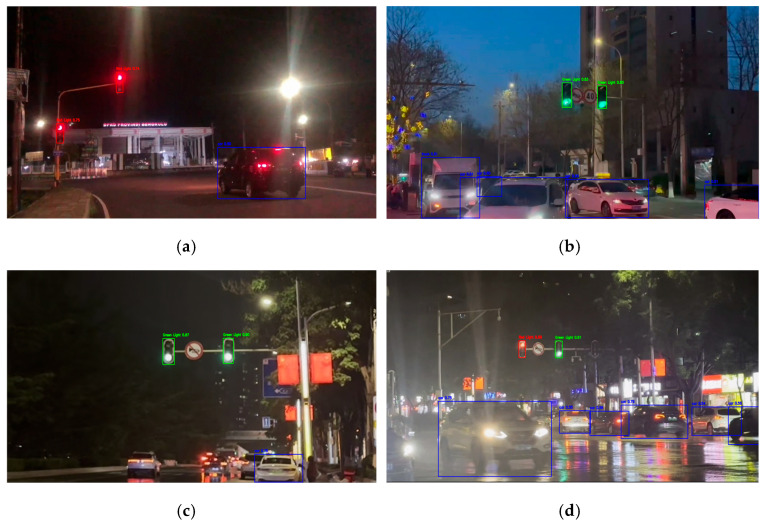
Nighttime testing scenarios. (**a**) Clear weather, Normal traffic (NCN); (**b**) Clear weather, Crowded traffic (NCC); (**c**) Rainy weather, Normal traffic (NRN); (**d**) Rainy weather, Crowded traffic (NRC).

Testing data are divided into daytime (Figure 4) and nighttime (Figure 5). Each time category was tested in different environmental conditions to determine the robustness and accuracy of the algorithm. In clear weather, natural lighting dominates, while in rainy weather, water droplets and reduced visibility interfere with detection performance. Both situations were further evaluated under normal and heavy traffic conditions, where vehicle density and obstacles can affect object visibility. A similar procedure was also applied to nighttime conditions. Nighttime testing was especially emphasized due to the difficulty of detecting traffic lights with dark or black frames in low-light conditions. In such scenarios, traffic light frames often blend into the background, leaving only the illuminated signals visible, which increases the risk of false detection or false negatives.

### 4.2. Computational Efficiency and Real-Time Performance

The combination of various test scenarios was planned to evaluate system performance under diverse traffic conditions, ranging from ideal sunlight to challenging low-light and wet environments. A quantitative summary of average accuracy (confidence), processing speed (FPS), and inference time for each condition is shown in Table 2, providing an objective basis for evaluating the computational efficiency of the proposed system.

We recorded an average frames per second (FPS) of 4.75, average inference time of 0.205 s, and average processing time of 0.214 s. The amount of data acquired is based on the hardware used, since all training and inference tasks were performed with a CPU.

### 4.3. Detection Accuracy and Model Robustness

Each of the eight environmental conditions is depicted in a 2–3 min video (totaling 18 min) comprising footage sourced from various locations. The algorithm’s detection performance for each dataset is shown in Table 3. The system was assessed under eight environmental conditions to thoroughly evaluate its performance based on these data. It should be noted that the reported average detection confidence reflects the model’s internal assurance during real-time operation and does not represent ground-truth-based accuracy.

The performance of this system was evaluated based on its usage environment, namely, on devices that only use CPUs. Due to variations in video duration, the number of recorded intersections, and traffic light cycle frequency, testing videos in different settings resulted in varying numbers of traffic light detections. For example, the Night–Clear–Normal (NCN) scenario produced the lowest number of detections at 9099, while the Day–Rain–Crowded (DRC) scenario recorded the highest number at 49,198. Higher detection counts were naturally associated with longer recordings and more frequent traffic light cycles.

The system’s real-time reliability was assessed using the average detection confidence level, calculated as the mean confidence score of all detected traffic light instances during operation. This provides a practical indicator of system assurance in live deployment, where ground-truth annotations are not accessible for immediate real-time comparison. The maximum confidence level observed achieved 0.95 across all detections, with an overall average detection confidence level of 0.73. The highest confidence levels were recorded during the day in clear weather and light traffic, while the lowest values were recorded in more complex scenarios, such as nighttime in crowded traffic.

### 4.4. Spatial Robustness and Signal Configuration Analysis

**Figure 6:**
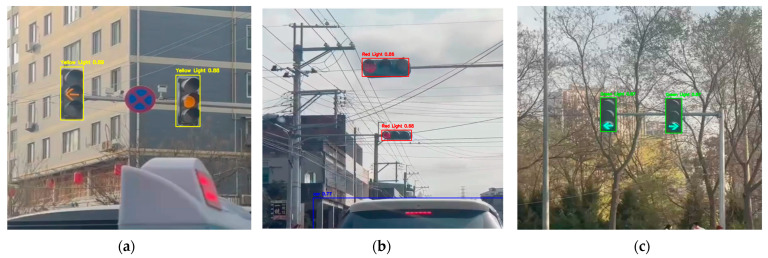
Detection performance across diverse configurations: (**a**) Vertical frame detection; (**b**) horizontal frame detection; (**c**) recognition of active status in arrow-shaped signals.

In addition to standard circular traffic lights, this system is evaluated based on its ability to recognize traffic lights with different orientations and shapes, such as horizontal configurations and directional signals. The system correctly interprets the active signal based on its position, as shown in Figure 6.

Critically, this system does not rely entirely on color information. It utilizes the spatial and structural configuration of traffic lights. During the detection process, the model recognizes the entire traffic light array, and the active signal is determined based on the relative position of the lit lights in the frame (top–middle–bottom for vertically arranged lights and left–middle–right for horizontally arranged lights).

The advantage of this system lies in its spatial positioning logic, rather than simply shape classification. By detecting the entire traffic light frame, this model identifies the active status of the signal based on its position relative to the frame. In horizontal configurations, the system correctly interprets active signals based on their position. The system points out exactly which part of the traffic light is active, effectively translating the arrow’s meaning through its position in the frame.

## 5. System Implementation and Application Design

This section describes the runtime implementation and functional behavior of the proposed assistive traffic light recognition system from a user-centered perspective. The application operates in real time on a CPU-only platform and provides both visual and auditory feedback without requiring an Internet connection.

**Figure 7:**
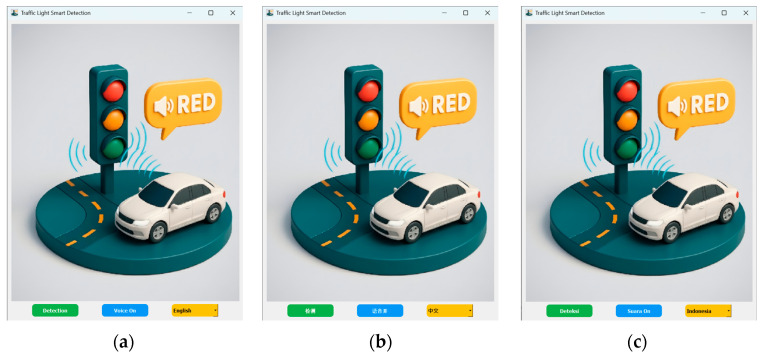
Initial Interface of the Traffic Light Smart Detection application. The application is presented in three language options: (**a**) English, (**b**) Mandarin, and (**c**) Indonesian.

The Traffic Light Smart Detection application integrates trained YOLO-based detection models, real-time visual output rendered through a QLabel-based interface, and offline multilingual audio instructions generated via a local text-to-speech engine. Figure 7 illustrates the graphical user interface of the application under different language configurations, demonstrating the system’s ability to adapt both textual and interaction elements according to user-selected languages.

**Figure 8:**
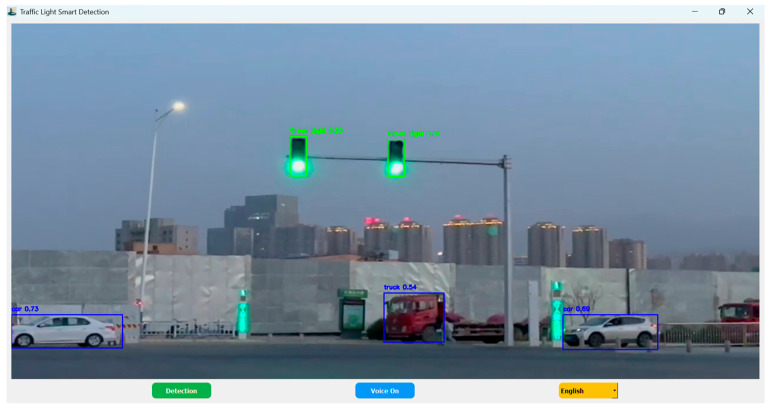
Application Runtime Detection.

When detection is activated, the application initializes a real-time video stream using OpenCV and processes incoming frames through a periodic inference loop controlled by a timer mechanism. This design ensures stable frame updates while maintaining a responsive graphical user interface, as illustrated in Figure 8. Each frame is preprocessed and forwarded to the trained YOLOv12 traffic light detection model to determine the current signal state. In parallel, a generic YOLOv12 model is used to detect surrounding objects relevant to traffic density analysis. Detection outputs include bounding boxes, class labels, and confidence scores, which are visualized in real time. Detection continues until the user terminates the process, at which point the video stream is safely released.

### 5.1. Visual Perception

The visual perception module processes video input captured from a vehicle-mounted camera and performs frame-by-frame inference in real time. To improve reliability and reduce visual clutter, only detections with confidence scores above 0.50 are retained. Detected traffic lights are highlighted using color-coded bounding boxes corresponding to the inferred signal state, while other detected objects are displayed using a neutral color scheme.

Confidence scores and object labels are displayed alongside each bounding box to provide transparent feedback regarding detection reliability. A periodic timer governs the inference cycle to ensure synchronized processing and consistent visualization. The interface is intentionally designed with a minimalist layout to avoid cognitive overload, allowing users to quickly interpret relevant information while driving.

### 5.2. Audio Feedback

The auditory feedback instructions will be delivered to the user based on detection results. This implementation uses a Python 3.12.7 library that enables local text-to-speech (TTS), allowing audio to be produced directly through the system speaker without requiring an Internet connection or external audio files. Warning messages for each traffic light condition are generated dynamically through the local TTS module (pyttsx3), using the computer’s operating system voice. The system generates distinct audio instructions corresponding to each light status as follows:

- Red Light: “Red light, please stop and relax!”
- Yellow Light: “Yellow light, please prepare!”
- Green Light: “Green light, you may go, have a pleasant journey!”

To reduce continuous repetition of sounds when frames are detected, audio is only activated when there is a change in traffic light signal detection, or from undetected to detected. As long as the light status does not change, even though the object remains detected in many consecutive frames, the sound is not played repeatedly. A minimum time limit of 1 s is also applied between sound playbacks. Even if there is a very rapid change in status, the system will wait for this time interval before allowing the next sound to play, reducing a user’s cognitive fatigue from becoming mentally tired due to excessive or repetitive information by overlapping sounds. Object detection continues to be displayed in real time, without slowing down due to sound output. This is because sound is processed in separate threads so that detection remains smooth.

### 5.3. Multilingual

To enhance inclusivity and usability across different regions, the application supports multilingual interaction in English, Mandarin, and Indonesian. Users can select their preferred language before or during system operation without restarting the application. The selected language consistently affects all interface elements, including textual labels, brief explanations, and audio feedback.

Each language option is paired with a corresponding voice configuration in the offline TTS engine, ensuring coherent and natural communication. By providing multilingual visual and auditory feedback, the system is designed to accommodate users from diverse linguistic and cultural backgrounds, particularly in regions with heterogeneous traffic regulations and driving environments.

## 6. Conclusions

### 6.1. Main Contributions

This study proposes and validates an innovative assistive system for drivers with color vision deficiency (CVD), centered on a spatial-position inference framework for traffic light recognition. By decoupling signal understanding from color perception, our work provides a robust technological intervention that contributes to the development of more inclusive and safety-oriented Intelligent Transportation Systems (ITSs). The experimental analysis yields the following key outcomes:

- After comparison with previous model training results, this study decided to use YOLOv12 for implementation in the user application;
- The model was tested with eight scenarios involving poor conditions at night, bad weather, and crowded traffic. During day testing, the system achieved an average detection confidence of approximately 0.73 with a maximum confidence level of 0.95, while at night, the detection performance decreases, especially in crowded environments;
- The application is designed with traffic light visualization that includes bounding boxes, name labels, and confidence levels. The audio produced followed the user’s language selection and is only played when there is a change in traffic light status.

### 6.2. System Limitations and Safety Considerations

Methods that rely entirely on color are prone to classification errors if the system is trained only through annotations on active lights without labeling the entire traffic light frame. By prioritizing the position and structure of active lights, the proposed system can reduce the risk of misinterpretation, including critical false negatives such as recognizing car backlights or street lights as traffic lights in nighttime conditions with heavy traffic density. The main problem is that the effectiveness of full detection depends on the vehicle’s camera. If the image input is poor or the resolution is low, the system will have difficulty capturing the full details of the traffic light structure. As a result, the risk of detection failure becomes higher.

In some conditions where red and yellow lights are lit simultaneously in the same frame of a traffic light, the system has difficulty determining signal priority, causing detection ambiguity. We still need to refine the algorithm so that it does not get confused when faced with such double signals. In more complex scenarios where false negatives may occur, such as Night–Rain–Crowded conditions, the absence of system output should be interpreted as a cue for increased driver vigilance rather than as an indication to proceed, reinforcing the role of the system as a supplementary assistance tool.

### 6.3. Future Work

Based on the results of this prototype, future development can focus on the model training process by analyzing the confusion matrix to evaluate detection errors during model training. The algorithm can be optimized to improve detection accuracy and speed. In addition, expanding the variety of datasets related to distance, position, size, and perspective of traffic lights is also very important. Further system development could combine the model with additional sensors, especially when dealing with extreme weather conditions and complex environments.

It is important to directly integrate the system into vehicles, including comparisons of the use of cameras inside or outside the car. The system also needs to support more flexible natural language settings, with additional language options for users from different backgrounds. A user-friendly interface design for individuals with color vision deficiency needs to be designed for ease of use. Finally, hands-on testing for users with color vision deficiency is necessary to ensure the effectiveness of the system.

## Figures and Tables

**Table 1:** Performance comparison of YOLO-based models on the same dataset.

Model	Precision	Recall	mAP@50	mAP@95
YOLO 8n	0.916	0.895	0.923	0.537
YOLO 10n	0.892	0.840	0.892	0.524
YOLO 11n	0.927	0.895	0.913	0.532
YOLO 12n	0.918	0.895	0.920	0.539

**Table 2:** Summary of average system performance in various scenarios.

Scenario	FPS	Inference	Process
DCN	4.639	0.211	0.219
DCC	4.634	0.211	0.220
DRN	4.623	0.212	0.221
DRC	4.555	0.215	0.224
NCN	4.908	0.197	0.205
NCC	4.991	0.193	0.201
NRN	4.913	0.198	0.205
NRC	4.758	0.205	0.213

**Table 3:** Detection performance metrics.

Metrics		Daylight				Night			
		Clear		Rain		Clear		Rain	
		Normal	Crowded	Normal	Crowded	Normal	Crowded	Normal	Crowded
Green Light Detected	Total	18,672	22,167	26,059	29,051	5456	9461	11,818	14,795
Red Light Detected	Total	10,379	14,166	15,785	16,372	2483	6235	6925	8066
Yellow Light Detected	Total	2306	2503	3542	3775	1160	1404	1692	2089
Green Light Detection Confidence	Avg	0.74	0.75	0.76	0.75	0.72	0.69	0.71	0.73
	Highest	0.95	0.95	0.95	0.95	0.91	0.91	0.91	0.92
Red Light Detection Confidence	Avg	0.74	0.74	0.74	0.74	0.73	0.70	0.71	0.72
	Highest	0.90	0.90	0.90	0.90	0.89	0.89	0.89	0.89
Yellow Light Detection Confidence	Avg	0.78	0.78	0.79	0.79	0.82	0.81	0.79	0.77
	Highest	0.91	0.91	0.91	0.91	0.91	0.91	0.91	0.91

## Data Availability

The datasets generated during this study are publicly available in the Zenodo repository at https://zenodo.org/records/18039880 (accessed on 7 January 2026).

## Abbreviations

The following abbreviations are used in this manuscript:

Avg	Average
CVD	Color Vision Deficiency
CNN	Convolutional Neural Network
DL	Deep Learning
FPS	Frames Per Second
GPU	Graphics Processing Unit
HSV	Hue, Saturation, Value
i7	Intel Core i7 Processor
mAP	Mean Average Precision
NCN	Night, Clear, Normal traffic scenario
NCC	Night, Clear, Crowded traffic scenario
NRN	Night, Rain, Normal traffic scenario
NRC	Night, Rain, Crowded traffic scenario
DCN	Day, Clear, Normal traffic scenario
DCC	Day, Clear, Crowded traffic scenario
DRN	Day, Rain, Normal traffic scenario
DRC	Day, Rain, Crowded traffic scenario
QTimer	Qt Timer (PyQt5)
SGD	Stochastic Gradient Descent
TTS	Text-to-Speech
YOLO	You Only Look Once
